# Effect of Different Water-to-Powder Ratios on the Compressive Strength of Calcium-enriched Mixture Cement

**DOI:** 10.22037/iej.v13i3.20568

**Published:** 2018

**Authors:** Nooshin Sadat Shojaee, Alireza Adl, Dana Jafarpur, Fereshte Sobhnamayan

**Affiliations:** a *Department of Endodontics, Dental School, Shiraz University of Medical Sciences, Shiraz, Iran; *; b *Department of Endodontics, Biomaterials Research Center, Dental School, Shiraz University of Medical Sciences, Shiraz, Iran; *; c *Dental School, Shiraz University of Medical Sciences, Shiraz, Iran*

**Keywords:** Calcium-enriched Mixture, CEM cement, Compressive Strength, Water-to-Powder Ratio

## Abstract

**Introduction:**

Calcium-enriched Mixture (CEM) cement is an endodontic reparative material available in the form of powder and liquid. The purpose of this *in vitro* study was to determine the effect of different water-to-powder (WP) proportions on the compressive strength (CS) of the cement.

**Method Materials and:**

One gram of CEM cement powder was mixed with either 0.33 g, 0.4 g or 0.5 g CEM liquid. The mixture was transferred to metallic cylindrical molds (*n*=10) with internal dimensions of 6±0.1 mm height and 4 ±0.1 mm diameter. After 4 days, the specimens were subjected to compressive strength tests using a universal testing machine. The data were analyzed by One-way ANOVA and Tukey’s tests at a significance level of 0.05.

**Results:**

Statically significant difference was found among experimental groups (*P*<0.05). The 0.33 WP ratio showed significantly greater CS value compared to 0.4 and 0.5 proportions (*P*=0.012 and *P*=0.000, respectively). The CS of 0.4 WP ratio was also significantly higher than that of 0.5 WP ratio (*P*=0.014).

**Conclusion:**

According to the results, higher WP ratios results in lower CS of the cement.

## Introduction

Calcium-enriched mixture (CEM) is a tooth-colored water-based cement which was first introduced to dentistry in 2006 as a novel endodontic cement [1]. The chemical composition of CEM cement is a mixture of different calcium compounds including, calcium oxide, calcium phosphate, calcium carbonate, calcium silicate, calcium sulfate, calcium hydroxide, and calcium chloride [2]. This cement has good handling characteristics, sets in an aqueous environment in less than 1 h, and forms an effective seal when used as a root-end filling material/apical plug [1, 3]. In addition, as a pulp capping agent, CEM cement has low tooth discoloration potential [4, 5].

The clinical applications of CEM cement are believed to be similar to those of mineral trioxide aggregate (MTA) [6]. Comparable results have been observed when MTA and CEM cement were used as pulp capping agent or furcation perforation repair [7, 8]. CEM cement has also shown favorable results regarding pulpotomy of permanent molar teeth with irreversible pulpitis [9], and management of internal root resorption [10]. Moreover, the antibacterial effect of CEM has been proved to be equal to calcium hydroxide and better than MTA [11] .

CEM is a powder containing fine hydrophilic particles that harden when they come in contact with a water-based solution. During and after mixing with its liquid, hydration reactions take place and calcium hydroxide is produced which is responsible for biologic properties of CEM cement [2]. Moreover, during hydration process other bioactive calcium and phosphate enriched materials are formed, which is compliant with the International Standard Organization (ISO) 6876 standard for dental root canal sealing materials [6, 12].

Like other hydroscopic cements, the water-to-powder (WP) ratio may affect the physical properties of this cement. Previous reports have suggested that higher WP ratios result in lower compressive [13] and push-out bond strength of MTA [14]. It has also been proposed that an increase in the WP ratio leads to a greater degree of MTA solubility and porosity [15]. 

As there is neither accurate manufacturer’s instruction nor any published study to recommend an appropriate WP ratio for mixing CEM cement, various ratios have been used in different studies. [16, 17]. Therefore, this in vitro study was conducted to assess the influence of WP ratio variations on the compressive strength of CEM cement.

## Materials and Methods

In this research, tooth-colored CEM cement (BioniqueDent, Tehran, Iran) was investigated. Primarily, the consistency of five different WP ratios (0.28, 0.33, 0.40, 0.50, and 0.60 grams of CEM liquid per gram of cement) were evaluated in a pilot study. The 0.6 ratio was not viscous enough for practical use and the 0.28 ratio was too dry to be manipulated. Therefore, the main study was conducted on 0.33, 0.40, and 0.50 WP ratios.

Six custom-made two-part split metal molds were used in the experiment. Each mold had five holes with internal diameter of 4±0.1 mm and thickness of 6±0.1 mm. All instruments and test materials were conditioned at 23^º^C± 1^º^C in the laboratory for 1 h prior to be used. The molds were randomly assigned into three groups according to different WP ratios. One gram of CEM powder was mixed with either 0.33 g, 0.4 g or 0.5 g CEM liquid (*n*=10). Immediately the mixtures were loaded incrementally into the holes by amalgam carrier and compacted with condensers with minimum pressure. 

The excess material was removed with wet cotton pellets. Wet pieces of gauze were placed on top and bottom of the molds and the specimens were then incubated at 37^°^C in 100% humidity.

After four days, the samples were removed from the incubator and the molds were split. The set CEM blocks were removed carefully by applying light force, taking care not to damage the CEM samples. After removal, the samples were visually evaluated for lack of voids or cracks. The samples were then submitted to compressive strength tests using a universal testing machine (ZwickRoell Group; Germany). The CEM blocks were placed lengthwise between the platens of the 

machine and compressed at a crosshead speed of 1 mm/min.

The maximum load needed to fracture each specimen was recorded and the compressive strength in mega Pascal (MPa) was calculated using the following formula: Compressive Strength (MPa) =4*p*/*πd*^2 ^, where *p* is the maximum load in Newton (N) and *d* is diameter of the specimens in mm.

One way ANOVA and Tukey’s HSD tests were used for statistical analysis. The significance level was set at 0.05.

## Results

The means and standard deviations of the compressive strength of different experimental groups are presented in Table 1. 

A statistically significant difference was found between groups (*P*=0.00). The highest compressive strength was observed in 0.33 WP ratio which was significantly different from those of 0.4 WP ratio and 0.5 WP ratio (*P*=0.012 and *P*=0.000, respectively). The 0.4 WP ratio also showed significantly greater compressive strength compared to 0.5 WP ratio (*P*=0.014).

## Discussion

This research was the first to evaluate the effect of WP ratio on the CS of CEM cement. CS is considered as one of the indicators of the setting and strength of hydraulic cements [18, 19] and may affect their clinical performance [20]. CEM cement is used in different clinical situations including perforation repair and vital pulp therapy; therefore, it should have sufficient CS to resist operative and functional loads [21, 22].

A variety of methods have been used to form cylindrical specimens of cements for compression testing. According to ISO 9917-1 (2003) standards, a split mold design, made of a material that will not be affected by the cement, has been advised [23]. In this experiment, stainless steel split molds were used. One of the advantages of split mold design is that the samples are removed easily with a light force [24].

A pilot study before the main research revealed that the WP ratio lower than 0.33 was too dry for practical use. At the same time, a 0.6 WP ratio was not viscous enough to be manipulated and 0.5 was the maximum WP proportion that allowed a mix of viscous consistency to be applied in practice.

The results of the present study demonstrated that CEM cement had higher CS in lower WP ratios. The maximum CS was obtained in the 0.33 WP ratio group which showed significant differences compared to 0.4 and 0.5 WP proportions.

Contact with a water-based solution is necessary for the hydration of CEM cement and expression of its biological and physical properties. During and after mixing with its liquid, calcium hydroxide is produced which dissociates into calcium and hydroxyl ions, increasing the pH and calcium concentration [2]. 

Additionally, in the presence of an aqueous environment, CEM cement releases calcium as well as phosphorus ions from indigenous sources, which lead to hydroxyapatite formation. Thus, it provides an extra seal at the interface of the material and cavity walls [25].

Therefore, one may assume that higher WP ratios might be beneficial. However, based on the results of the present study, the excessive amount of water incorporated in the mix leads to a lower CS. In a study investigating the solubility and porosity of MTA using different WP ratios, Fridland and Rosado [15] reported that the porosity of MTA increased as the WP ratios increased. Therefore, lower compressive strength of CEM cement in the samples with higher WP ratio may be attributed to the higher porosity. However, as there is no published study on the effect of different WP ratio on the porosity of CEM cement, further studies are recommended on this subject.

**Table 1:** Mean (SD) (Mpa) of compressive strength of CEM recorded for each set of specimens with different water to powder ratios

**Water to powder ratio**	**Compressive strength**
0.33	1.32 (0.20)^A^
0.4	0.92 (0.41)^B^
0.5	0.53 (0.18)^C^

* *Different upper case letters show significant difference between compressive strength of different ratios*

It is noteworthy to mention that the same results have been reported for Pro Root MTA and Angelus MTA [13]. One of the limitations of the present research was that the effect of different WP ratios was evaluated only on the CS of CEM cement. As in a previous work, WP ratio affected the solubility and porosity of ProRoot MTA, further studies are recommended to evaluate the effect of different WP ratios on other physical properties of CEM Cement.

## Conclusion

Within the limitations of this *in vitro* study, it can be concluded that higher WP ratios lead to lower compressive strength of CEM Cement.
